# Hospital Airships

**Published:** 1919-07-19

**Authors:** 


### HOSPITAL AIRSHIPS.

In an interesting article in the June number of the *National Review*, Capt. E. Brown, R.A.M.C., calls attention to the possibilities of airships as purveyors of climate. Climate is almost as much a matter of altitude as of latitude, and by means of an airship any altitude may be quickly and comfortably attained.

In a general way, increasing altitude means an increase in the actinicity of the sunlight, with an increase in the rarity, asepticity, and dryness of the air, and each of these increases has its own therapeutic significance. The precise hygienic value of the actinic rays of the sun it would be difficult to estimate, but we know, among other things, that the actinic rays (at the violet end of the spectrum) kill many germs, increase absorption of oxygen by the tissues, and hasten the hatching of flies' and of frogs' eggs. We know, too, that it is not the actinic rays, but the rays at the red end of the spectrum that cause heat apoplexy, and it is pretty certain that, at ordinary Alpine altitudes, the actinic rays have a stimulating effect on the physiological functions of the body.

### The Treatment of Phthisis.

We do not agree with Capt. Brown that "the dead and stagnant air that lurks in the remoter recesses of the lungs" is "one of the main hindrances to a cure" [of phthisis] and would be got rid of by respiration in rarefied air. Respiration in rarefied air causes increased elimination of carbon dioxide from the blood, and from the air vesicles of the lung; but the carbon dioxide, a respiratory stimulant, is not deleterious, and we do not understand what else Capt. Brown can mean by "dead and stagnant air." We also question Capt. Brown's suggestion that breathing in rarefied air would benefit emphysematous conditions of the lungs, for the deepened inspiration with increased aspiration must nullify any benefit derived from lowered atmospheric pressure. On the other hand, there can be little doubt that the deepened breathing with increased aspiration tends to improve the nutrition of the lungs and their flexibility and power as organs of respiration. By frequent alterations of altitude, too, say from 1,000 ft. to 10,000 ft., it might be possible to massage, as it were, the lungs.

The danger of hæmorrhage from the lungs at ordinary altitudes is small, for at Alpine resorts, such as St. Moritz, hæmorrhages are not more frequent than on the plains. A rapid ascent doubtless puts strain on the pulmonary blood-vessels, but the strain is probably relieved to a great extent by the facilitation of circulation through the lungs by the increased expansion of the chest. It has long been known that residence at high altitudes promotes multiplication of the red blood cells, and possibly altitude may turn out to have curative effects on ordinary anæmia. The asepticity of the air at high altitudes is also of therapeutical importance. At great altitudes, as Tyndall and Pasteur showed, germs are absent and meat does not putrefy; at high health resorts cases of consumption avoid the danger of mixed infection, and no doubt wounds would heal readily without risk of suppuration. Even more important than the asepticity of air at high altitudes is its dryness. Half the water vapour of the atmosphere is below 7,000 feet and about three-quarters of it below 13,000 feet. Not only so, but the rarity of the air gives it more drying power. In the Engadine meat dried in the air is a national dish. The dryness and rarity of high air make it unable to retain heat, and so it cools rapidly when the sun leaves it; and this causes great differences in the temperature during the day and the night.

### The Therapeutical Advantages.

All the therapeutical advantages, then, to be derived from actinic light and from rare, aseptic, dry air could be quickly obtained by rising in an airship to any required height; and Capt. Brown believes that airship hospitals are quite practicable. Practicable they probably are, but there would be difficulties. At high altitudes there are apt to be strong winds, and though movement of air is physiologically healthful, yet we imagine that patients in airship hospitals would be exposed to more wind than is good for them, unless indeed the airship were to go before the wind, and that might have geographical disadvantages, for a gale might blow for a week in one direction, and a nervous patient might find himself one day over Greenland's icy mountains or India's coral strands. In any case, the difficulties of providing food and water would be considerable, and the difficulty of providing exercise almost insurmountable.

What would be the cost of running an airship with or against the wind for weeks or months we cannot guess, but petrol and oil and wages would amount to a goodly sum, and a hospital airship accordingly would not be a hospital for the poor. Nevertheless, despite such limitations and difficulties as we have indicated, hospital airships might perform many useful functions, and Capt. Browne has done well in drawing attention to their possibilities.

